# Supplementary material for: Detection rate and treatment gap for atrial fibrillation identified through screening in community health centers in China (AF-CATCH): A prospective multicenter study
Source: PLoS Med. 2020 Jul 16;17(7):e1003146. doi: 10.1371/journal.pmed.1003146 (PMC7365395; doi:10.1371/journal.pmed.1003146)
Supplement: S1 Text — (DOCX) [file pmed.1003146.s002.docx]

**Supplemental materials on the description of the health system in China**

China health system is predominantly public, which includes primary care service offered by community health centers and specialized service offered by secondary and tertiary hospitals.

1. **Community health centers in China**

**Function:** The Community Health Center in China is organized by the government, which provides non-profit primary care service for community residents, schools and pension institutions in the area.

It provides community residents with public health services such as disease prevention and basic medical services including diagnosis and treatment of common diseases, management of diagnosed chronic diseases, and rehabilitation medical services. It is responsible for the management of common chronic diseases of the elderly in the area, such as hypertension and diabetes, but right now atrial fibrillation is not yet on the list.

**Setting:** The Community Health Center is set up in principle in accordance with the jurisdiction of the sub-districts (townships). Each sub-district or township should have at least one community health service center. Sub-districts or townships with a population greater than 100,000 may have more than one community health service centers.

A community health center is usually composed of several departments, such as preventive medicine, general internal medicine, traditional Chinese medicine, rehabilitation medicine, medical laboratory, and radiology and ultrasound imaging.

**Personnel:** The employees of a Community Health Center include general practitioners, public health physicians, Chinese medicine practitioners (including integrated Chinese and Western medicine) and other licensed physicians, registered nurses, and qualified personnel of pharmacy, laboratory, imaging and other related health technologies.

**Medicare reimbursement:** The Community Health Center has a higher reimbursement rate from public health insurance than secondary and tertiary hospitals.

**Community health centers in Shanghai:** Shanghai has 18 districts. The number of community health centers in each district varies from 5 to 49 according to the number of residents. With a total of 215 administrative divisions:107 sub-districts, 106 towns, and 2 townships, there are 250 community health centers in the whole city of Shanghai. The community health center service in Shanghai is the best established in China.

The community health centers can provide residents with low-cost, nearby, convenient and fast medical and health services for common, frequently-occurring and chronic diseases. It is the usual place of the local residents, especially the elderly residents, to access primary care in urban areas.

1. **Secondary and tertiary hospitals**

A secondary hospital provides comprehensive medical and health service to several communities in a region and undertakes some teaching and research tasks. In general, county-level (countryside) and district-level (municipal) hospitals are usually at this second level. Such hospitals often have no less than 100 beds. The main responsibility of the secondary hospitals is to participate in the guidance of the monitoring of high-risk patients, and receive direct referrals from and provide technical guidance to community health centers. Secondary hospitals should be able to conduct some teaching and research work. Across Shanghai there are over 100 secondary hospitals including general hospitals, specialized hospitals (for instance, dentistry), factory or other staff hospitals, geriatric hospitals and psychiatric health centers.

A tertiary hospital provides medical and health services across regions and provinces, and is a medical center with comprehensive medical, teaching, and scientific research capabilities. It often has no less than 500 beds. The main responsibility of the tertiary hospitals is to provide specialized medical services, treat critical and complicated diseases, accept secondary referrals, provide technical guidance and personnel training for secondary hospitals, nurture health professionals, and undertake tasks of scientific research. Across Shanghai there are 38 tertiary hospitals. And over 20 tertiary hospitals have specialized cardiovascular clinics, 10 of which also have a specialized atrial fibrillation clinic.
