# Supplementary material for: Detection rate and treatment gap for atrial fibrillation identified through screening in community health centers in China (AF-CATCH): A prospective multicenter study
Source: PLoS Med. 2020 Jul 16;17(7):e1003146. doi: 10.1371/journal.pmed.1003146 (PMC7365395; doi:10.1371/journal.pmed.1003146)
Supplement: S3 Text — (DOCX) [file pmed.1003146.s004.docx]

***AF Questionnaire***

Centre number:□□□□ Subject ID:□□□□ Initials:□□□□ Gender：□M □F

Date of birth: Date of Screening:

**Do you have atrial fibrillation or atrial flutter before?** □Yes □No □ atrial flutter □atrial fibrillation

Onset time: hospital: disease document:

Type： □paroxysmal □persistent □unclear

**Do you have rheumatic heart disease**? □Yes □No If yes, Cardiac valve replacement? □Yes □No

**Do you on any drugs therapy？** □Yes □No

□Antiarrhythmic agents 1. propafenone dose/day 2. amiodarone dose/day 3. Other drugs

□Rate control 1.Digoxin dose/day 2. β-blocker dose/day

□Oral anticoagulants 1. Warfarin dose/day 2. Dabigatran dose/day 3. Other drugs

□Antiplatelet drugs 1. Asprin dose/day 2. Clopidogrel dose/day 3. Dual anti-platelet drugs

**Other drugs?**

Drug name dose/day Drug name dose/day Drug name dose/day

1 2 3

**Other therapy?**  Ablation □Yes □No Left Atrial Appendage Occlusion □Yes □No

**Do you have any disease as showed below?**

**Stroke or TIA:** □Yes □No Type：□cerebral Infarction □cerebral Hemorrhagic □TIA

Onset time: hospital: disease document:

**Thromboembolism:**□Yes □No Type：□gastrointestinal tract □kidney □ leg □lung □other

Onset time: hospital: disease document:

**Vascular disease:**□Yes □No Type：□MI □peripheral artery disease □aortic plaque □Other，please describe :

Onset time: hospital: disease document:

**Congestive Heart failure：**□Yes □No First diagnosed time： hospital: EF： %

**Hypertension**：□Yes □No First diagnosed time : Antihypertension drugs : □Yes □No

Drug name dose/day Drug name dose/day Drug name dose/day

1 2 3

**Diabetes**： □Yes □No First diagnosed time : Treatment : □Yes □No

Diet control：□Yes □No Oral hypoglycemic drugs：□Yes □No Insulin injection：□Yes □No

FBG: mmol/L 2-hour postprandial blood glucose: mmol/L glycosylated hemoglobin: %

**If you have any other disease, please describe** :

**Present habit?**

**Current smoking**: □Yes □No cigarette amount : /day age of starting smoking

If not now, age of quit smoking

**Habitual drinking:** □Yes □No age of starting drinking alcohol consumption :

□ spirit : amount: ml /day days /week □ yellow rice wine: amount: ml /day days /week

□ beer: amount: ml /day days /week □ red wine: amount: ml /day days /week

If not now, age of quit drinking

**Physical Examination**

Weight: kg Height: cm Waist circumferences: cm hip circumferences: cm

Arm selected : □left □right Cuff size: □Normal □Large Device: _________

Sitting BP measurement: SBP1/DBP1 / mmHg HR _____/min

SBP2/DBP2 / mmHg HR _____/min

SBP3/DBP3 / mmHg HR _____/min

**ECG:**  Device: ________ Diagnosis this time: □Atrial fibrillation □Not Atrial fibrillation

Time of next visit: Investigator signature:
