# Supplementary material for: Detection rate and treatment gap for atrial fibrillation identified through screening in community health centers in China (AF-CATCH): A prospective multicenter study
Source: PLoS Med. 2020 Jul 16;17(7):e1003146. doi: 10.1371/journal.pmed.1003146 (PMC7365395; doi:10.1371/journal.pmed.1003146)
Supplement: S4 Text — (DOCX) [file pmed.1003146.s005.docx]

***AF Follow-up Questionnaire***

Centre number:□□□□ Subject ID:□□□□ Initials:□□□□ Gender：□M □F

Date of birth: Date of Screening:

**Living status：**Live die If died，death time / / ，cause of death：

1．Main cause of death：

2．Direct cause of death：

3．Other cause of death：

**Has there been any new illness since the last follow-up? If so, please describe**

Disease 1: _____________ Onset time: hospital: clinical outcome

Disease 2: _____________ Onset time: hospital: clinical outcome

**Are there any other adverse events since the last follow-up? If so, please describe**

Adverse event 1: ______________ Onset time: details

Adverse event 2: ______________ Onset time: details

**Have you attended any AF specialist clinic? □Yes □No**

**If yes, time: hospital: department**

**What drug therapy have you received？**

□Antiarrhythmic agents 1. propafenone dose/day 2. amiodarone dose/day 3. Other drugs

□Rate control 1.Digoxin dose/day 2. β-blocker dose/day

□Oral anticoagulant 1. Warfarin dose/day 2. Dabigatran dose/day 3. Other drugs

□Antiplatelet drugs 1. Asprin dose/day 2. Clopidogrel dose/day 3. Dual anti-platelet drugs

**Other drugs?**

Drug name dose/day Drug name dose/day Drug name dose/day

1 2 3

**Other therapy?**  Ablation treatment □Yes □No Left Atrial Appendage Occlusion □Yes □No

**If no, why didn’t you attend the AF specialist clinic?**

**Physical Examination**

Weight: kg Height: cm Waist circumferences: cm hip circumferences: cm

Arm selected: □ left □ right Cuff size: □ Normal □ Large Device: _________

Sitting BP measurement: SBP1/DBP1 / mmHg HR _____/min

SBP2/DBP2 / mmHg HR _____/min

SBP3/DBP3 / mmHg HR _____/min

**ECG:** Device: ________ Diagnosis on this time: □ Atrial fibrillation □ Not Atrial fibrillation

Investigator signature:
