# Supplementary material for: Detection rate and treatment gap for atrial fibrillation identified through screening in community health centers in China (AF-CATCH): A prospective multicenter study
Source: PLoS Med. 2020 Jul 16;17(7):e1003146. doi: 10.1371/journal.pmed.1003146 (PMC7365395; doi:10.1371/journal.pmed.1003146)
Supplement: Attachment — Submitted filename: Response_to_reviewer.docx [file pmed.1003146.s006.docx]

| Comments: | Reply |
| --- | --- |
| Editor： |  |
| 1. Title- Please revise your title according to PLOS Medicine's style. Your title must be nondeclarative and not a question. It should begin with main concept if possible. "Effect of" should be used only if causality can be inferred, i.e., for an RCT. Please place the study design ("A randomized controlled trial," "A retrospective study," "A modelling study," etc.) in the subtitle (ie, after a colon). | **As requested, we have revised the title as followed:**  *“Detection rate and treatment gap for atrial fibrillation identified through screening and an education program in community health centers in China (AF-CATCH): a prospective cross-sectional study.”* |
| 2. At this stage, we ask that you include a short, non-technical Author Summary of your research to make findings accessible to a wide audience that includes both scientists and non-scientists. The Author Summary should immediately follow the Abstract in your revised manuscript. This text is subject to editorial change and should be distinct from the scientific abstract. Please see our author guidelines for more information: [https://journals.plos.org/plosmedicine/s/revising-your-manuscript#loc-author-summary](https://journals.plos.org/plosmedicine/s/revising-your-manuscript#loc-author-summary) | An author summary has been inserted as requested:  **AUTHOR SUMMARY**  **Why was this study done?**   - Atrial fibrillation (AF) is a common heart rhythm problem that often has no symptoms, so it is often underdiagnosed - People with AF can have a very high stroke risk which is highly preventable with appropriate oral anticoagulant (OAC) medications - The prevalence of unknown and untreated AF in the Chinese community is not known, nor whether patient education in the community health center has the potential to improve OAC prescription.   **What did the researchers do and find?**   - We screened for AF in residents aged ≥65 years in community health centers in Shanghai, and provided disease education and advice on referral to specialist clinics for OAC treatment to people with newly detected or undertreated known AF. - We demonstrated a noticeable gap in AF detection and treatment: 2.8% of those screened had unknown or untreated AF. - At 12-months, only 17/103 people with newly detected or undertreated known AF attended specialist clinics, and only 4/17 had commenced OAC therapy.   **What do these findings mean?**   - We highlight a serious public health issue in China with under-diagnosis and under-treatment of AF in the community that requires a whole of system approach. - To prevent AF-related stroke in China, greater efforts must be made to increase appropriate OAC therapy in people with AF. |
| 3. Abstract- please clarify that all patients had a single timepoint screen rather than repeated screens for AF? This point is pertinent for paroxysmal AF perhaps and that a single point screening is possibly an underestimate | As suggested, we have amended the abstract for clarity:  **Abstract, Methods and findings section:**  *“We performed a single timepoint screening for AF with a handheld single-lead ECG in Chinese residents (≥65 years) in five community health centers in Shanghai from April to September 2017.”* |
| 4. Abstract methods and findings- the last sentence should be a limitation of your study design. If the lack of OAC initiation is a reflection of the healthcare system in China, this should not be listed as a limitation but in the conclusion section. | As suggested, we have amended this section to include this limitation:  **Abstract, Methods and findings section:**  *“As screening was performed at a single timepoint, some paroxysmal AF cases may have been missed, thus the rate of new AF may be underestimated.”* |
| 5. Abstract- you note that only 17 out of 103 patients attended speciality clinics. Can you clarify if these individuals were not referred to a speciality centre or if they did not attend of their own accord? This will impact how you frame the conclusions | We have amended the findings for clarity as follows:  **Abstract, Methods and findings section:**  *“At 12-months follow-up in 103 patients (81% complete), despite disease education and advice on specialist referral, only 17 attended specialist clinics and 4 were prescribed OAC. Of those not attending specialist clinics,* *71 chose instead to attend community health centers or secondary hospital clinics with none prescribed OAC, and 15 had no review.”* |
| 6. Line 2- is atrial fibrillation a growing problem? This implies that the incidence has increased. If this is true please provide a reference to support this and also reword for clarity | We have modified the manuscript as follows:  **Introduction, paragraph 1:**  *“Atrial fibrillation (AF) is a growing problem in cardiovascular disease, with age-adjusted incidence rates on the rise [1]. It is predicted that AF prevalence will at least double in the next 30 years[2,3]. Patients with AF have about 5-fold increased risk of ischemic stroke [4,5], which is highly preventable with appropriate oral anticoagulant (OAC) therapy [6].”* |
| 7. Methods-could you name the participating hospitals here? Shanghai is a bit state, you may want to highlight the participating communities via a map | As suggested, we have amended the manuscript to list the participating communities and have included a map of these areas as a figure.  **Methods, Study Population, paragraph 1:**  *“Our study subjects were residents aged 65 years or older recruited from five community health centers (Yuyuan Community Health Center, Laoximen Community Health Center, Ruijin 2^nd^ Road Community Health Center, Puxing Community Health Center, Sanlin Community Health Center) in urban areas of Shanghai from April to September 2017 (Fig 1). A description of the community health centers in China is provided in the S2 Text.”*   Fig 1. Location of the five communities:  *Left: Map of Shanghai City; Right: Location of the five communities located within Shanghai City.* |
| 8. Did your study have a prospective protocol or analysis plan? Please state this (either way) early in the Methods section. a) If a prospective analysis plan (from your funding proposal, IRB or other ethics committee submission, study protocol, or other planning document written before analyzing the data) was used in designing the study, please include the relevant prospectively written document with your revised manuscript as a Supporting Information file to be published alongside your study, and cite it in the Methods section. A legend for this file should be included at the end of your manuscript. b) If no such document exists, please make sure that the Methods section transparently describes when analyses were planned, and when/why any data-driven changes to analyses took place. c) In either case, changes in the analysis-- including those made in response to peer review comments-- should be identified as such in the Methods section of the paper, with rationale. | To address this important point, we have added a paragraph at the beginning of the methods section to clarify that the protocol and statistical analysis plan were previously published. We have provided more details of the education intervention in the methods section. We have also referred to the previously published statistical analysis plan and included it as a reference [15].  **Methods, Paragraph 1:**  *This study was a prospective cross-sectional study conducted in five communities (Yuyuan Community, Laoximen Community, Ruijin 2^nd^ Road Community, Puxing Community, Sanlin Community) in urban areas of Shanghai from April to September 2017. This study was part of a larger study (AF-CATCH, CT02990741) and the protocol has been previously published in detail [15]. The study was approved by the Ethics Committee of Ruijin Hospital, Shanghai Jiaotong University School of Medicine. Our study was conducted in accordance with the principles of the Declaration of Helsinki. This study is reported as per the Strengthening the Reporting of Observational Studies in Epidemiology (STROBE) guideline (S1 Checklist).*  **Methods, “Screening and** **Intervention” section, paragraph 3:**  *“All participants not receiving guideline recommended OAC were deemed ‘actionable AF’ (including both known AF and newly detected AF) and were included in our educational intervention program. The education program included one-on-one disease education with the research cardiologist at the community health center and provision of educational materials. Education included AF being associated with high risk of stroke, the appropriate treatment options and benefits and risks of anticoagulants.”*  **Methods, “statistical analysis” section:**  *“Statistical analyses were performed in accordance with our statistical analysis plan using SAS 9.3 (SAS Institute Inc., Cary, NC, USA) [15]”* |
| 9. Please ensure that the study is reported according to the STROBE guideline, and include the completed STROBE checklist as Supporting Information. Please add the following statement, or similar, to the Methods: "This study is reported as per the Strengthening the Reporting of Observational Studies in Epidemiology (STROBE) guideline (S1 Checklist)." The STROBE guideline can be found here:  [http://www.equator-network.org/reporting-guidelines/strobe/](http://www.equator-network.org/reporting-guidelines/strobe/)  When completing the checklist, please use section and paragraph numbers, rather than page numbers. | As requested, we have ensured that the manuscript is reported according to the STROBE guideline, have included the completed guideline, and have added the STROBE reporting statement.  **Methods, paragraph 1:**  *"This study is reported as per the Strengthening the Reporting of Observational Studies in Epidemiology (STROBE) guideline (S1 Checklist)."* |
| 10. Methods- please provide a copy of the medical history, lifestyle questionnaire used | As requested, we have included copies of the questionnaires as supplements:  **Methods, “Screening and Intervention” section, paragraph 2:**  *“A questionnaire regarding medical history, lifestyle and use of medications was administered to all participants by the research cardiologists (**S3 Text).”*  **Methods, “Screening and Intervention” section, paragraph 3:**  *“For patients with AF history or AF rhythm, CHA_2_DS_2_-VAS_c_ score was calculated to estimate the risk of stroke and determine eligibility for OAC according to the 2016 ESC guidelines (i.e. CHA_2_DS_2_-VASc ≥ 2 men or ≥ 3 women: Class-1 OAC recommendation) [5]. Details of current antithrombotic treatment was confirmed and recorded using the patients’ medical record (S4 Text).”*  **Methods, “Follow-up” section,** **paragraph 1:**  *“A follow-up questionnaire was administered by the research cardiologist regarding living status, adverse outcomes including stroke and myocardial infarction in the past 1 year, whether they attended the AF specialist clinic or not, what treatment they received from specialists especially in relation to OAC prescription, and if relevant, why they did not attend the AF specialist clinic (S5 Text).”* |
| 11. Methods- please do not use the phrase “elderly subjects” as 65 years and over constitutes a large age range | As suggested, we have rephrased our use of the term “elderly subjects” throughout the manuscript. |
| 12. Abstract, introduction and methods- it is unclear if the educational “intervention” should be renamed since this is not a trial? Please also clarify if the education and advice was given to patients directly or given to community health centre staff. | We provided an educational intervention at the time of screening and again at one month, with the hypothesis that provision of education and referral to a specialist clinic would increase prescription of OAC. The study outcome therefore was the number prescribed OAC over the course of the study. We have improved the clarity of reporting in our manuscript to address this point, and to clarify to whom the education was provided.  **Abstract, Introduction, methods and findings:**  *“Disease education and advice on referral to specialist clinics for OAC treatment was provided to all patients with actionable AF (newly detected or undertreated known AF) at the time of screening, and education was reinforced at 1-month.”*  **Methods, Screening and Intervention, paragraph 2-3:**  *For patients with AF history or AF rhythm, CHA_2_DS_2_-VAS_c_ score was calculated to estimate the risk of stroke and determine eligibility for OAC according to the 2016 ESC guidelines (i.e. CHA_2_DS_2_-VASc ≥ 2 men or ≥ 3 women: Class-1 OAC recommendation) [5]. Details of current antithrombotic treatment was confirmed and recorded using the patients’ medical record (S4 Text). All participants not receiving guideline recommended OAC were deemed ‘actionable AF’ (including both known AF and newly detected AF) and were included in our educational intervention program. The education program included one-on-one disease education with the research cardiologist at the community health center and provision of educational materials. Education included AF being associated with high risk of stroke, the appropriate treatment options and benefits and risks of anticoagulants. Because warfarin, non-Vitamin K oral anticoagulants, and INR testing are not available at community health centers, patients were advised to attend a specialist clinic for review and OAC prescription. Information on how to make an appointment at a designated specialist clinic near each community was also provided. Across Shanghai there are over 20 tertiary hospitals with specialist cardiovascular clinics, 10 of which also have a specialist AF clinic. These tertiary hospitals are located between 2-10 kms from the community health centers, and provide the majority of OAC prescription and INR testing for the region.*  *At 1-month, attendance at the specialist clinic was confirmed via telephone. If they had not attended, we invited them to return for review and another single-lead ECG at the community health center. Education and advice on specialist referral was reinforced.*  **Methods, Follow-up section:**  *Follow-up occurred at 12-months in the community health centers. A follow-up questionnaire was administered by the research cardiologist regarding living status, adverse outcomes including stroke and myocardial infarction in the past 1 year, whether they attended the AF specialist clinic or not, what treatment they received from specialists especially in relation to OAC prescription, and if relevant, why they did not attend the AF specialist clinic (S5 Text). If participants were unable to attend a visit in the community health center, telephone follow-up was offered.* |
| 13. Results- please provide details of how many patients at the community health centre were approached, how many declined to participate. | As requested, we have added further details as follows:  **Results, “Characteristics of the study population” section**  *“It is estimated that the 5 participating community health centers service a total of 9,710 aged 65 years and over each year. During the study period 4,784 residents attended the clinic and were approached to participate in the study, and 253 declined (response rate 94.7%). A total of 4531 citizens (2530 women [55.8%]; mean (± SD) age 71.6 ± 6.3 years) participated in the screening program.”* |
| 14. Throughout- please use lower case p for p- values | As requested, we have changed the Capital P to lower case p throughout the manuscript. |
| 15. Throughout- where you mention ESC guidelines- please ensure you introduce ESC on first view | **As suggested, we introduced ESC guidelines in the introduction, and summarized again in the methods:**  **Introduction, last sentence:**  *“……to improve OAC prescription in patients with ‘actionable AF’ (i.e., those identified as newly diagnosed AF or undertreated known AF) who have a Class-1 recommendation for OAC thrombo-prophylaxis according to the 2016 European Society of Cardiology (ESC) guidelines (i.e. CHA_2_DS_2_-VAS_c_ score ≥ 2 in men or ≥ 3 in women) [5] .”*  **Methods, Screening and Intervention, paragraph 2:**  *“For patients with AF history or AF rhythm, CHA_2_DS_2_-VAS_c_ score was calculated to estimate the risk of stroke and determine eligibility for OAC according to the 2016 ESC guidelines (i.e. CHA_2_DS_2_-VASc ≥ 2 men or ≥ 3 women: Class-1 OAC recommendation) [5].”* |
| 16. Line 108-113 this information should be provided much sooner and more clearly. Please also provide additional details about the education provided at baseline. | As suggested, additional information on the educational intervention has been included in the methods section:  **Methods, Screening and Intervention, paragraph 2:**  *For patients with AF history or AF rhythm, CHA_2_DS_2_-VAS_c_ score was calculated to estimate the risk of stroke and determine eligibility for OAC according to the 2016 ESC guidelines (i.e. CHA_2_DS_2_-VASc ≥ 2 men or ≥ 3 women: Class-1 OAC recommendation) [5]. Details of current antithrombotic treatment was confirmed and recorded using the patients’ medical record (S4 Text). All participants not receiving guideline recommended OAC were deemed ‘actionable AF’ (including both known AF and newly detected AF) and were included in our educational intervention program. The education program included one-on-one disease education with the research cardiologist at the community health center and provision of educational materials. Education included AF being associated with high risk of stroke, the appropriate treatment options and benefits and risks of anticoagulants. Because warfarin, non-Vitamin K oral anticoagulants, and INR testing are not available at community health centers, patients were advised to attend a specialist clinic for review and OAC prescription. Information on how to make an appointment at a designated specialist clinic near each community was also provided. Across Shanghai there are over 20 tertiary* *hospitals with specialist cardiovascular clinics, 10 of which also have a specialist AF clinic. These tertiary hospitals are located between 2-10 kms from the community health centers, and provide the majority of OAC prescription and INR testing for the region.* |
| 17. Conclusion- I would probably avoid the phrase "big gap" as this is subjective. | As suggested, we have amended the conclusions  **Conclusions, first sentence:**  *“There is a noticeable gap in the detection and treatment of AF in the Chinese* residents aged 65 and over *in urban Shanghai.”* |
| 18. PLOS Medicine requires that the de-identified data underlying the specific results in a published article be made available, without restrictions on access, in a public repository or as Supporting Information at the time of article publication, provided it is legal and ethical to do so. Please see the policy at  [http://journals.plos.org/plosmedicine/s/data-availability](http://journals.plos.org/plosmedicine/s/data-availability) and FAQs at [http://journals.plos.org/plosmedicine/s/data-availability#loc-faqs-for-data-policy](http://journals.plos.org/plosmedicine/s/data-availability#loc-faqs-for-data-policy) | Data Availability Statement:  Our de-identified data has been attached as a supplementary document(S6 Data). |
| 19. Throughout- please provide 95% CI along with p-values | We have provided the 95% confidence intervals as suggested.  **Methods, statistical analysis section:**  *“New episodes of AF were expressed as true positives divided by the total number screened with accompanying binomial 95% confidence intervals (CIs) calculated using Clopper–Pearson methodology.”*  **Results, Characteristics of the study population section, paragraph 1:**  *“The prevalence of AF was 4.0% (95% CI, 3.5% - 4.7%) (n = 183), including 1.8% (95% CI, 1.4% - 2.2%) with ECG-confirmed known AF (n = 82), 1.7% (95% CI, 1.4% - 2.2%) with known AF in sinus rhythm (n = 79), and 0.5% (95% CI, 0.3% - 0.7%) with ECG-confirmed previously unknown AF (n = 22).”* |
| Reviewer #1: |  |
| 1. One could question the role of ECG screening in patients with known AF. If a patient is known to have AF and has not initiated OAC, why would another AliveCor ECG documenting AF help? | Performing the 30-second handheld screen was an opportunity to review the current rhythm status of the patients, especially for those with paroxysmal AF. We agree that for the patients with known AF, the additional screening does not change the diagnosis, however it does provide an educational opportunity to initiate the conversation around AF, what it means and why OAC is required. As we were referring these patients to a specialist clinic for review for OAC, it was important to provide education and for patients to understand what AF is. |
| 2. Consider adding this citation to your Discussion: Heart Rhythm. 2019 Aug;16(8):e59-e65. | **We have added the citation as suggested:**  **Discussion, paragraph 6:**  *“Innovative approaches involving current mobile and wireless technologies to record multiple ECG snapshots with patient-activated handheld single-lead ECG devices may help improve the detection rate [25]”* |
| Reviewer #2: |  |
| 1. Line 76: "For all analyses, a two-sided probability value < 0.05 was considered statistically significant." In the statistical jargon, it is actually referred to as a two-sided "p-value". | We have amended the manuscript as suggested  **Methods, Statistical Analysis section:**  *“For all analyses, a two-sided p-value < 0.05 was considered statistically significant.”* |
| 2. Line 98: "OAC medication rate was 20% in all patients with known AF at baseline (28/138 ESC guideline recommended for OAC)". Can I just clarify that I understand the numbers correctly? There are 161 patients with known AF, and 183 patients with AF in total. 155/183 had CHA2DS2-VASc score >= threshold. Then out of the 161 with known AF, 138 were guideline recommended and only 28 were taking AOC? Figure 2 is excellent by the way and all answers are there, but perhaps you could you provide the breakdown of the 155 in the text so that it's easier when you read and don't have the Figure in front of you. | **As suggested, we have modified the manuscript to improve clarity:**  *“The majority of the AF patients (155/183, 85%) had a class-1 recommendation for OAC therapy [5]: specifically, 138/161 (86%) known AF and 17/22 (77%) new AF (Fig 3). Of the OAC eligible patients, 11.0% (17/155) had a HAS-BLED Score ≥ 3, and 4 patients with known AF had previously stopped warfarin because of bleeding (2 retinal hemorrhage, 2 hematuria). OAC prescription rate (for those with a class-1 recommendation) was 20% (28/138) in known AF patients and 0% in new AF patients (Fig 3).”* |
| 3. Line 115: "Follow-up occurred in 126/155 patients at 12-months". Did you do the follow up only in the 155 patients who had CHA2DS2-VASc score >= threshold? If yes, could you please clarify in the Methods why this is the case? | As the 12-month follow-up was aimed at all 183 patients, we have amended this section for clarity.  **Results, 12-month Follow-up section:**  *“Follow-up occurred in 148/183 patients at 12-months (81% complete): 96 in-person at the community health center and 52 by telephone. From the OAC eligible patients, 126/155 (81%) were followed up, and 29 were lost to follow up (21 unable to contact, 4 declined a phone interview, 2 had hearing difficulty and lived alone, and 2 had moved and were not available) (Fig 3).”* |
| 4. Line 119: there is a typo in the word "stroke" that is currently spelled as "stoke". | Thank you. We have reviewed and corrected the typos throughout the manuscript. |
| Reviewer #3: |  |
| 1. I do think that this manuscript could benefit from English language editing | As suggested. we have edited the manuscript to improve the grammar and use of English Language |
| 2. Abstract-Methods and findings    You should be clear in the abstract that it was a single measurement for AF. | In response to this comment and Editor Comment 3, we have amended the abstract for clarity:  **Abstract, Methods and findings section:**  ***“****We performed a single timepoint screening for AF with a handheld single-lead ECG in Chinese residents (≥65 years) in five community health centers in Shanghai from April to September 2017.****”*** |
| 3. Abstract-I don't agree with your statement that "The main limitation of our study is that the investigators were not able to provide patients who needed OAC therapy with either warfarin or non-warfarin oral anticoagulants .." This is not a limitation of your study, but a limitation of your healthcare system and should be framed as such. | In response to this comment and Editor Comment 4, we have amended this section:  **Abstract, Methods and findings section:**  *“As screening was performed at a single timepoint, some paroxysmal AF cases may have been missed, thus the rate of new AF may be underestimated.”* |
| 4. Study population-Can you please describe exhaustively and precisely how the program was advertised? Was it only by notice to the neighborhood committee and by placements of posters? If so, this sentence should read "The screening program was publicized through official notices to the neighborhood committee and placement of posters in community health centers." The current wording makes it sound like there were other channels | As suggested, we have improved the description for how the screening program was advertised.  **Methods, study population section:**  *“The screening program was publicized through a public health press conference and media release in Shanghai, official notices of the neighborhood committee, and placement of posters in community health centers.”* |
| 5. Screening and intervention-Can you provide more description of the community health clinic? This would help with the generalizability and reproducibility off the study. Is it physician run? Are there other health professionals? Is this the usual place that study participants access primary care or is this more of a drop in? | As requested, we have included further information on the Community health clinic setting in China.  **Methods, study population section:**  *“A description of the community health centers in China is provided in the S2 Text.”* |
| 6. Screening and intervention-Rather than state that the ECGs were reviewed by an "investigator" or by a "the first author", this should be framed in the context of qualifications. Was it an arrhythmia specialist, cardiologist who reviewed them? | As recommended, we have clarified the profession of the person reviewing the ECGs  **Methods, Screening and Intervention section, paragraph 1:**  *“Each ECG rhythm strip was reviewed by a* *cardiologist from the research team (CY) at the screening visit. The ECGs were classified into three groups: sinus rhythm, AF and uninterpretable. Participants with uninterpretable single lead ECG were referred for 12-lead ECGs, which were reviewed by* *a second cardiologist (WD or WJG).”* |
| 7. Please provide a citation for OAC guidelines. | We have reviewed the manuscript and ensured that a citation is provided for each mention of the 2016 ESC guidelines. |
| 8. Can you provide a more detailed description of the educational intervention? Was there a standard script? Did you use any patient decision-making tools? Was it done by a physician or other health care professional? How did you re-contact them at 1 month? It may be reasonable to provide a general overview in the methods and a detailed overview in an appendix. | As requested, we have revised the description of the education intervention and supplied the specific questionnaires used as supplement material.  **Methods, Screening and Intervention section, paragraphs 2-3:**  *For patients with AF history or AF rhythm, CHA_2_DS_2_-VAS_c_ score was calculated to estimate the risk of stroke and determine eligibility for OAC according to the 2016 ESC guidelines (i.e. CHA_2_DS_2_-VASc ≥ 2 men or ≥ 3 women: Class-1 OAC recommendation) [5]. Details of current antithrombotic treatment was confirmed and recorded using the patients’ medical record (S4 Text). All participants not receiving guideline recommended OAC were deemed ‘actionable AF’ (including both known AF and newly detected AF) and were included in our educational intervention program. The education program included one-on-one disease education with the research cardiologist at the community health center and provision of educational materials. Education included AF being associated with high risk of stroke, the appropriate treatment options and benefits and risks of anticoagulants. Because warfarin, non-Vitamin K oral anticoagulants, and INR testing are not available at community health centers, patients were advised to attend a specialist clinic for review and OAC prescription. Information on how to make an appointment at a designated specialist clinic near each community was also provided. Across Shanghai there are over 20 tertiary* *hospitals with specialist cardiovascular clinics, 10 of which also have a specialist AF clinic. These tertiary hospitals are located between 2-10 kms from the community health centers, and provide the majority of OAC prescription and INR testing for the region.*  *At 1-month, attendance at the specialist clinic was confirmed via telephone. If they had not attended, we invited them to return for review and another single-lead ECG at the community health center. Education and advice on specialist referral was reinforced.* |
| 9. Can you describe the AF specialist clinics better? How many are there? Are they near the community health centres? Is the only place in the region where anticoagulants are prescribed? In most other countries, family physicians or generally practitioners are competent and comfortable with OAC prescription. | As raised by the reviewer, there are some differences in China surrounding how OAC is prescribed, therefore we have provided further information on specialist clinics and community health centers in the methods.  **Methods, Screening and Intervention, Paragraph 2:**  *“Because warfarin, non-Vitamin K oral anticoagulants, and INR testing are not available at community health centers, patients were advised to attend a specialist clinic for review and OAC prescription. Information on how to make an appointment at a designated specialist clinic near each community was also provided. Across Shanghai there are over 20 tertiary hospitals with specialist cardiovascular clinics, 10 of which also have a specialist AF clinic. These tertiary hospitals are located between 2-10 kms from the community health centers, and provide the majority of OAC prescription and INR testing for the region.”* |
| 10. Results-The results section should begin with some broader statement of the eligible population. This is vital to understand the uptake of screening and the subset of the population that did participate.  Can you provide some estimate of the number of potentially eligible persons in the cachement area of the clinic? Perhaps you have census data or some other estimate of the roster of patients at the community clinics? | As suggested, we have re-written this section with the requested information.  **Results, characteristics of the study population section, paragraph 1:**  *“It is estimated that the 5 participating community health clinics service a total of 9,710 aged 65 years and over each year. During the study period 4,784 residents attended the clinic and were approached to participate in the study, and 253 declined (response rate 94.7%). A total of 4531 citizens (2530 women [55.8%]; mean (± SD) age 71.6 ± 6.3 years) participated in the screening program.”* |
| 11. The statement "Because warfarin, non-Vitamin K oral coagulants, and INR testing are not available at these community health centers, patients were advised to attend specialist clinics for OAC prescription with information on how to make an appointment." Does not belong in the results. It should be in the methods, with further detail as per my comment above. | As recommended, we have moved this information to the methods section  **Methods, Screening and Intervention, paragraph 2:**  *“The education program included one-on-one disease education with the research cardiologist at the community health center and provision of educational materials. Education included AF being associated with high risk of stroke, the appropriate treatment options and benefits and risks of anticoagulants. Because warfarin, non-Vitamin K oral anticoagulants, and INR testing are not available at community health centers, patients were advised to attend a specialist clinic for review and OAC prescription. Information on how to make an appointment at a designated specialist clinic near each community was also provided. Across Shanghai there are over 20 tertiary* *hospitals with specialist cardiovascular clinics, 10 of which also have a specialist AF clinic. These tertiary hospitals are located between 2-10 kms from the community health centers, and provide the majority of OAC prescription and INR testing for the region.”* |
| 12. Discussion-I think your discussion is too long. I would aim to shorten it by at least 33% | We have rewritten the discussion in order to reduce the overall length by 33%, and make the discussion more concise, taking into consideration the below comments 13-15.  **Discussion**  *The main findings of our study are that AF is prevalent in Chinese people aged 65 years and older, with an overall prevalence of 4.0%, and that actionable AF constituted 2.8% of all those screened, a very high proportion in whom initiation of OAC therapy could make a difference to prognosis. Of those with known AF qualifying for guideline recommended OAC prophylaxis, only 20% were taking OAC. The percentage was even lower (17%) when patients with valvular prostheses requiring warfarin were excluded, highlighting the significant under-treatment of AF in patients managed by community health centers. Ischemic stroke or death occurred in 5 patients (none were taking OAC) in 1 year, potentially preventable if guideline recommended OAC had been used.*  *Our data highlight a serious public health issue in China, with under-treatment of AF in the community which is likely to result in an excess of preventable stroke and death. Our OAC treatment rates are lower than those reported in Chinese tertiary hospitals (9.6% to 68.4%) and nontertiary hospitals (4.0% to 28.2%) [16], however our study may more closely reflect a real-world sample of the community-dwelling population aged ≥65 years in China. Despite providing education regarding AF and advice on specialist referral for all 127 patients with actionable AF, the majority did not attend a specialist clinic, and of the 17 who attended the specialist clinic only 4 were commenced on OAC therapy.*  *Low prescription of OAC is complex and compounded by many factors, including: a) patient reluctance to attend as they did not consider themselves very ill despite one-on-one education; b) patients elected to attend their local community health center and believed their AF was adequately treated, even though they were only prescribed antiplatelet drugs and traditional* *Chinese medicine; and c) community health center physicians may lack knowledge regarding evidence-based management of AF. Furthermore, there was also low adherence to AF guidelines by the specialists in the tertiary hospital clinics which may be the result of a) worries about patient treatment compliance to warfarin in the absence of a dedicated anticoagulant clinic or team in the local area, b) the common misperception of aspirin efficacy and safety [17,18]; c) restricted use and/or unavailability of the new oral anticoagulants (NOAC) even in some tertiary hospitals; and d) the expense of NOACs may prohibit patient agreement to the prescription.*  *Education for both patients, and physicians in the community health centers, is required to overcome these barriers. It has been demonstrated, in five countries, that multifaceted education programs targeting both patients and providers can significantly increase the proportion of patients treated with OAC from 68% to 80% at 1 year [19]. In China, it may be possible to run workshops providing simplified important information on AF. Workshops for patients should focus on disease education to improve health-seeking behaviors, whereas physician education should focus more on understanding evidence-based treatment for AF, and establishment of direct and efficient referral pathways for patients to access appropriate treatment with a cardiologist. Directed health resource allocations for patients to access anticoagulant drugs and INR testing in community health centers, such as a regular specialist outreach clinic, may help enhance adherence and long-term persistence with OAC. Furthermore, public health policies that include NOACs into medical insurance will give both patients and doctors more alternatives to choose from [20].*  *Designated pathways to treatment are very important if AF screening is to be undertaken. In AF screening studies it is well documented that the success for guideline adherence to OAC treatment is influenced by the pathway to treatment offered within the study. In the Swedish STROKESTOP study, an OAC review with the study cardiologist was part of the screening process and 99% were reviewed, resulting in 74% of people with actionable AF having OAC initiated [21]. However, the Belgium Heart Rhythm Week Study which screened in the community and had a similar pathway to our study (i.e. providing advice for patients to consult their general practitioner or cardiologist) found that only 11.2% of eligible participants with actionable AF commenced OAC therapy [22]. Recently, the Huawei Heart Study [23] in China reported ~80% of OAC-eligible AF patients were anticoagulated, using a programme of integrated AF management directed by a mobile AF application. However, in the Huawei Heart Study there is a likely selection bias as participants all owned smartphones, were able to download the app, and they chose to use smart wearable devices to monitor their own pulse rhythm. The cohort was much younger, and likely had higher health literacy and interest in improving their health and therefore were more likely to respond to physician advice.*  *Our finding of a detection rate of 0.5% new AF, is comparable to the 0.5 - 0.8% identified in other mass community screening programs using a single-time screen [21,24]. However we detected fewer cases of new AF than we anticipated, compared to the result of 2.0% identified in 2011 in our previous study [13]. The lower detection rate may be related to the fact that 82% of our participants had undergone an ECG in the past two years, as part of the free annual health examinations (including a 12-lead ECG) in community health centers for people ≥65 years, which have been organized and supported by the Chinese government over the past five years. This may support the notion that annual ECG checks indeed help to detect AF early. However, annual ECG checks or single-time handheld ECG screens may still underestimate the prevalence of AF, as cases paroxysmal AF may not be captured. Innovative approaches involving current mobile and wireless technologies to record multiple ECG snapshots with patient-activated handheld single-lead ECG devices may help improve the detection rate [25]. This was demonstrated in the STROKESTOP Study, where 0.5% new AF was identified with the first screen, and after 2 weeks of intermittent twice-daily screening, the yield of new AF increased to 3% [21]. The REHEARSE-AF study also identified a higher yield of 3.8% using 1 to 2 ECG recordings per week over one year [26]. However, before more frequent or intensive screening for unknown AF could be recommended in China, greater effort must be made to increase OAC treatment in known AF.*  *There are some limitations to our study that warrant discussion. First, the screening was performed at a single timepoint, and some cases of paroxysmal atrial fibrillation may have been missed, thus the detection rate of AF may be underestimated. Second, despite our strict follow-up procedures the loss to follow up was 19.1%. Third, our study protocol did not allow the research cardiologist to directly review and treat patients with OAC, however this reflects the real-world pathway to treatment for community-based Chinese. Finally, we did not involve physicians in the community health centers into our screening and education program to encourage initiation of and adherence with OAC, which may also contribute to the low rate of referral.* |
| 13. It is interesting that among patients with actionable AF "only 17 attended cardiovascular specialist clinics and 4 of these were prescribed OAC" That is a discouragingly low rate and suggests that there is another critical barrier at the level of the specialist clinic. This should be re-inforced and discussed. | We have addressed this point as part of the extensive revision of the discussion outlined above |
| 14. You postulate reasons why patients did not attend specialist clinics, but only one of these are supported directly by the findings of your study or by references. You also postulate possible interventions that could help improve OAC adherence. This paragraph has the same issues. These two paragraphs could be combined, shortenend and amended. | We have addressed this point as part of the extensive revision of the discussion outlined above |
| 15. I don't think that the paragraph that begins "Our community study found significant under-treatment of known AF in sinus rhythm … is particularly useful. Given that you took only a single ECG and you don't have the power to do comparative statistics between the 3 groups, it is largely an over-interpretation of your data and it distracts from your overall message. | We have addressed this point as part of the extensive revision of the discussion outlined above |
