# Supplementary material for: Detection rate and treatment gap for atrial fibrillation identified through screening in community health centers in China (AF-CATCH): A prospective multicenter study
Source: PLoS Med. 2020 Jul 16;17(7):e1003146. doi: 10.1371/journal.pmed.1003146 (PMC7365395; doi:10.1371/journal.pmed.1003146)
Supplement: Attachment — Submitted filename: Response_to_reviewers.doc [file pmed.1003146.s007.doc]

| Comments: | Reply |
| --- | --- |
| **Editors’ comments：** |  |
| 1. Title- the title currently indicates the education program was carried out in community health centres. Please consider revising to “Detection rate and treatment gap for atrial fibrillation identified through screening in community health centers in China (AF-CATCH): a prospective cross-sectional study” | In response to this comment and Reviewer 3 Comment, we have revised the title as follows:  *“Detection rate and treatment gap for atrial fibrillation identified through screening in community health centers in China (AF-CATCH): a prospective multi-center study.”* |
| 2. Abstract Please add brief participant demographics | As requested, we have added brief participants demographics to the abstract:  *“The 183 patients with AF were older, taller, and more likely to be male, and had a higher prevalence of cardiovascular disease than those without AF (p<0.02).”* |
| 3. Methods INR testing? please define on first view | We have reviewed the manuscript and ensured that the definition of INR is provided on first view.  **Methods, Screening and Intervention section, paragraph 2:**  *“Because warfarin, non-Vitamin K oral anticoagulants, and international normalized ratio (INR) testing are not available at community health centers, patients were advised to attend a specialist clinic for review and OAC prescription.”* |
| 4. Informed consent-please mention details of consent within the methods section | As requested, we have added details of consent to the methods section:  **Methods, Study Population, paragraph 1:**  *“All participants were informed of the study design and gave their written informed consent before joining the screening program. The consent included agreements to share their information for confidential academic analysis and agreements with the follow-up arrangements in the study.”* |
| 5. Please also mention any additional follow-up after the 12 month follow-up. If patients were still not on OACs but have AF, were they referred to a specialist clinic again? | As suggested, information on the additional follow-up has been included in the methods section:  **Methods, Follow up, paragraph 1:**  *“If patients had not attended a specialist clinic within the 12-month period, AF education and the importance of attending a designated AF specialist clinic was reinforced.”* |
| 6. Table1 – p-values to three decimal places is sufficient | As suggested, we have modified p-values over three decimal places to “<0.001” in Table 1. |
| 7. The excel file containing de-identifying data contains data that may breach patient confidentiality such as date of birth. Please remove this information for privacy reasons and deposit these files with your local research ethics committee instead. The data availability statement should be revised to note that there are ethical restrictions on data sharing. Also, at this point please amend the statement in your methods section “All the records and information on participants were anonymized and de-identified before the analysis” as these do not appear to be de-identified medical records. | As requested, we have removed the excel file from the supplemental materials, and in response to the comment in “Production issues, METADATA 7”, we revised the data availability statement as follows:  “*Data cannot be shared publicly because of ethical restrictions. Data are available from Ruijin Hospital Ethics Committee (contact via wyfkjc@163.com) for researchers who meet the criteria for access to confidential data.*”  We have also deleted the following statement in the methods section:  “*All the records and information on participants were anonymized and de-identified before the analysis.*” |
| 8. STROBE checklist- please check the provided checklist and corresponding paragraph numbers as they do not match the main text. For instance, you say example the discussion is in paragraph 7, but it's not. Please correct and clarify this. | Thank you. We have reviewed the checklist and ensured that the corresponding paragraph numbers match the main text. |
| **Reviewer #1:** |  |
| While my concerns remain about the incremental contribution beyond previous screening studies and the lack of success in initiating anticoagulation, the authors have made an extensive effort to revise this manuscript and address all reviewer comments. | We appreciate your time and comments. |
| **Reviewer #2:** |  |
| This is a statistical review of manuscript PMEDICINE-D-19-04467_R1. I thank the authors for their answers for my previous comments, which are satisfactory. I do not have further comments. | We appreciate your time and comments. |
| **Reviewer #3:** |  |
| Thank you for allowing me to review a revised version of your manuscript. You have satisfactorily addressed all of the comments I raised in my first review and you have submitted a greatly improved paper.  I have one final comment that stems from a change you made between the original submission and first submission. Your title describes the study as a prospective, cross-sectional study. **Prospective** is correct, but **cross-sectional** is not. **Prospective** refers to measuring an exposure at baseline (in your case AF) and then an outcome (OAC use) at some point in the future. **Cross-sectional** would mean you measured them both at a single time point. | In response to this comment and Editor Comment 1, we have revised the title as follows.  *“Detection rate and treatment gap for atrial fibrillation identified through screening in community health centers in China (AF-CATCH): a prospective multi-center study.”* |
| **Production issues:** |  |
| 1. Please provide exact p-values in the abstract and throughout the manuscript, unless p<0.001. Please note we do not permit instances of p<0.05. | We have reviewed the manuscript and ensured that the p-values provided meet the editorial requirements. |
| **FIGURES and GRAPHICS:**  1. Please note that your figures do not currently comply with our publication requirements. For further instructions to assist with your Figure revisions, please refer to our Figure Guidelines at: <http://journals.plos.org/plosmedicine/s/figures.>  If you need assistance, please contact us directly at [graphics@jjeditorial.com](mailto:graphics@jjeditorial.com) so we can further help with this matter. Please send any relevant source files and tell us what application(s) and what operating system you are using.  - The text in Figure 1 is below our recommended font size (8-12 pt) and difficult to read. Please remake the text in question at a larger size or take steps to make the text legible. If the text is irrelevant to the figure's meaning, please tell us and we will publish the figure as is.  - Figures 2 and 3 are acceptable for publication. | We downloaded the two maps of Figure 1 (Left: Map of Shanghai City; Right: Map of Shanghai Downtown area.) from website. The font of the text in the map changes as the map size changes.  We marked location of our five participating communities in the map to show that they are all in urban areas of Shanghai. The text in the map (Figure 1) is irrelevant to the figure's meaning, we think it’s OK to publish the figure as it is. |
| 2. Please include the location from which you obtained the base map used in Figure 1 in the legends for this figure. Please note that, because all PLOS articles are published under a CC BY license ([creativecommons.org/licenses/by/4.0/](http://creativecommons.org/licenses/by/4.0/)), we cannot publish proprietary maps such as Google Maps, Mapquest or other copyrighted maps. If your map was obtained from a copyrighted source please amend the figure so that the base map used is from an openly available source.  The following websites are good examples of where you can source open access or public domain maps: * World of Maps - Many, but not all, of the maps are in the public domain so please check the usage rights before downloading. ([http://www.worldofmaps.net](http://www.worldofmaps.net/)) * U.S. Geological Survey (USGS) - All maps are in the public domain. ([http://www.usgs.gov](http://www.usgs.gov/)) * PlaniGlobe - All maps are published under a Creative Commons license so please cite “PlaniGlobe, http://www.planiglobe.com, CC BY 2.0” in the image credit after the caption. (<http://www.planiglobe.com/?lang=enl>) * Natural Earth - All maps are public domain. (<http://www.naturalearthdata.com/about/terms-of-use/>) | We downloaded the two maps of Figure 1 (Left: Map of Shanghai City; Right: Map of Shanghai Downtown area.) from website below, which is an openly available source.  Shanghai Platform for Common Geospatial Information Services <http://shanghai.tianditu.gov.cn/map/views/standardMap.html>  And as suggested, information has been included in the legend of Figure 1.  *“Maps were obtained from* [*http://shanghai.tianditu.gov.cn/map/views/standardMap.html*](http://shanghai.tianditu.gov.cn/map/views/standardMap.html)*”* |
| 3. Please remove embedded figures from your manuscript file and ensure the most up to date versions of your figures are uploaded to our submission system as separate TIF or EPS files. Please do not remove the figure legends and captions. | Completed. |
| 4. Please ensure that all main figure files are cited in ascending numerical order in the main text of the article. | Completed. |
| 5. Please ensure that all main figures are referenced in the manuscript as Fig 1, Fig 2, etc. (including capitalization), rather than Figure 1, Figure 2, etc. Please note, however, that the file names themselves must not include the space, i.e. Fig1.tif, Fig2.tif, etc. | Completed. |
| 6. Please ensure that all main figure legends are placed after the paragraph containing their first in-text citation. | Completed. |
| **TABLES:** 1. Table 2 uses returns to control text wrapping. This is indicated by a [¶ or ↵] symbol when "Show Paragraph Marks" is turned on in Word. Please either split those cells into separate cells to achieve alignment or remove the returns. | As suggested, we split those cells into separate cells. |
| **METADATA:**  1. Please ensure that the CRediT author contributions listed for each co-author are completed in full. To qualify for authorship, each author must meet at least one of the seven core contributions (conceptualization; data curation; formal analysis; investigation; methodology; software; validation), as well as at least one of the writing contributions (original draft preparation; review and editing). Authors may also be noted against any of the remaining contributions, however these alone will not qualify a contributor for authorship.  Please ensure that the contributions listed on the submission form for each author qualify them for authorship based on the above criteria. Please ensure that the full contributions of each author are acknowledged in the "Add/Edit/Remove Authors" section of our submission form. If they do not meet criteria for authorship please let us know as soon as possible by emailing [plosmedicine@plos.org](mailto:plosmedicine@plos.org). | Completed. |
| 2. Please ensure only the authors' initials are included in the competing interest statement, not their full names or titles. | Completed. |
| 3. Please provide a detailed Financial Disclosure statement on the submission form in full sentences, and include URLs, the initials of the receiving author, and specific grant numbers wherever possible. | Completed. |
| 4. Please ensure that the funders and grant numbers match between the Financial Disclosure field and the Funding Information tab in your submission form. Note that the funders must be provided in the same order in both places as well. | Completed. |
| 5. In accordance with PLOS' data policy, please ensure that your Data Availability Statement in the submission form clearly identifies how readers can access your data. Data cannot be available on request, or only accessible by contacting one of the authors. Note that your Data Availability Statement will be typeset as it is written, so please ensure it is in complete sentences and appears as you would like it to in the published version.  For details on our data policy, please see <http://journals.plos.org/plosmedicine/s/data-availability> | As requested by editor’s comment 7 above, we have removed our excel file from the supplemental materials, and in response to the comment in “Production issues, METADATA 7”, we revised the Data Availability Statement as follows:  “*Data cannot be shared publicly because of ethical restrictions. Data are available from Ruijin Hospital Ethics Committee (contact via wyfkjc@163.com) for researchers who meet the criteria for access to confidential data.*” |
| 6. In the online submission form, you indicated that "Data cannot be shared publicly because of ethical restrictions. Data are available from Ruijin Hospital Ethics Committee (contact via [qifanghuang@163.com](mailto:qifanghuang@163.com)) for researchers who meet the criteria for access to confidential data." All PLOS journals now require all data underlying the findings described in their manuscript to be freely available to other researchers, either 1. In a public repository, 2. Within the manuscript itself, or 3. Uploaded as supplementary information.  This policy applies to all data except where public deposition would breach compliance with the protocol approved by your research ethics board. If your data cannot be made publicly available for ethical or legal reasons (e.g., public availability would compromise patient privacy), please explain this by return email and staff will assist you with completing your Data Availability Statement.  At this time, we ask that you please explain, with as much detail as possible, why you are unable to make your data available in one of the three places mentioned above so we can further assess whether or not this complies with PLOS’ Data Policy and determine how we can assist you in this matter. | As requested by editor’s comment 7 above, we have removed our excel file from the supplemental materials, and in response to the comment in “Production issues, METADATA 7”, we revised the Data Availability Statement as follows:  “*Data cannot be shared publicly because of ethical restrictions. Data are available from Ruijin Hospital Ethics Committee (contact via wyfkjc@163.com) for researchers who meet the criteria for access to confidential data.*” |
| 7. Please note that the contact for accessing your data cannot be an author on the manuscript. Please therefore replace the current contact information with a 3rd party contact, such as an institutional data access committee. | As requested, we replaced the current contact information with a 3rd party contact as follows:  “*Data cannot be shared publicly because of ethical restrictions. Data are available from Ruijin Hospital Ethics Committee (contact via wyfkjc@163.com) for researchers who meet the criteria for access to confidential data.*” |
| **ARTICLE FILE:**  1. Please indicate the corresponding author in the byline by placing an asterisk (*) after their affiliation number.  2. Please include the corresponding author's email address on the title page of your manuscript, indicated by an asterisk (*). Only an asterisk and the email address itself are required.  3. Please remove author degree titles from the byline of your article file.  4. Please remove the funding information from your manuscript file. The published version is pulled automatically from the “Financial Disclosure” field in the submission form and must not be duplicated. Please therefore take this opportunity to check that the information in this field is correct and completed in full.  5. Please remove the Competing Interests section from your manuscript file. The published version is pulled automatically from the "Competing Interests" field in the submission form and must not be duplicated. Please therefore take this opportunity to check that the information in this field is correct and completed in full.  6. Please ensure that your Ethics statement is available in the Methods section of your manuscript in its entirety. | Completed. |
| **REFERENCES:**  1. Please ensure your references are in the style of PLOS Medicine (ICMJE/Vancouver style). The reference style guide can be found here: [http://journals.plos.org/plosmedicine/s/submission-guidelines#loc-references.](http://journals.plos.org/plosmedicine/s/submission-guidelines#loc-references.) You can also find a downloadable EndNote file in these guidelines to assist you. Please do a manual check as well, as the EndNote file does not catch everything. Please note that the references will be typeset as they appear so please ensure the format is consistent.  2. Please put all reference citations in square brackets separated by either commas or dashes with no spaces, e.g. [1,2,3] or [1-3].  3. Please make sure all references are cited in ascending numerical order in the text. | Completed. |
| **SUPPORTING INFORMATION:**  1. Please ensure that all Supporting Information files use the following format verbatim throughout the manuscript (including legends and all in-text citations): S1 Fig, S1 Table, S1 Text, S1 Data, etc. Each file type should start at 1 and increase numerically. Please note, however, that the file names themselves must use an underscore rather than a space, i.e. S1_Fig.tif, S1_Table.xlsx, S1_Text.doc, S1_Data.xlsx, S1_PRISMA_Checklist.docx, etc.  2. Please ensure that each supporting information file has a legend at the end of the manuscript file, after the Reference list.  3. Please note that your submission cannot contain any references to generic "supporting information" files and instead must refer to specific files using our naming convention (S1 Fig, S1 Table, S1 Text, S1 Data, SI PRISMA Checklist, etc.) | Completed. |
| **Production issues 2:** |  |
| EDITORIAL:  1. In the abstract you have stated: "The 183 patients with AF were older, taller, and more likely to be male, and had a higher prevalence of cardiovascular disease than those without AF (p < 0.02)". Please provide the exact p-values in all cases, except when p<0.001. | As requested, we provided the exact p-values :  *“The 183 patients with AF were older (p < 0.001), taller (p = 0.02), and more likely to be male (p = 0.01), and had a higher prevalence of cardiovascular disease* *than those without AF (p < 0.001).”* |
| FIGURES and GRAPHICS:  1. Please note that your figures do not currently comply with our publication requirements. For further instructions to assist with your Figure revisions, please refer to our Figure Guidelines at: <http://journals.plos.org/plosmedicine/s/figures.>  If you need assistance, please contact us directly at [graphics@jjeditorial.com](mailto:graphics@jjeditorial.com) so we can further help with this matter. Please send any relevant source files and tell us what application(s) and what operating system you are using.  - The text in Figure 1 is still below our recommended font size (8-12 pt) and difficult to read. Please remake the text in question at a larger size or take steps to make the text legible. If the text is irrelevant to the figure's meaning, please tell us, and we will publish the figure as is.  - Figures 2 and 3 are acceptable for publication.  2. Unfortunately, the source for the map used in Figure 1 does not meet our requirements for copyright licensing and cannot be published. Please note that, because all PLOS articles are published under a CC BY license ([creativecommons.org/licenses/by/4.0/](http://creativecommons.org/licenses/by/4.0/)), we cannot publish proprietary maps such as Google Maps, Mapquest or other copyrighted maps.  The following websites are good examples of where you can source open access or public domain maps: * U.S. Geological Survey (USGS) - All maps are in the public domain. ([http://www.usgs.gov](http://www.usgs.gov/)) * PlaniGlobe - All maps are published under a Creative Commons license so please cite “PlaniGlobe, http://www.planiglobe.com, CC BY 2.0” in the image credit after the caption. (<http://www.planiglobe.com/?lang=enl>) * Natural Earth - All maps are public domain. (<http://www.naturalearthdata.com/about/terms-of-use/>) | We remade Figure 1 based on public geographic information, and we hope this will comply with the publication requirements.   |
| METADATA:  1. Please ensure that the funders and grant numbers match between the Financial Disclosure field and the Funding Information tab in your submission form. Note that the funders must be provided in the same order in both places as well. | Completed. |
| SUPPORTING INFORMATION:  1. Please ensure that all Supporting Information files use the following format verbatim throughout the manuscript (including legends and all in-text citations): S1 Fig, S1 Table, S1 Text, S1 Data, etc. Each file type should start at 1 and increase numerically. Please note, however, that the file names themselves must use an underscore rather than a space, i.e. S1_Fig.tif, S1_Table.xlsx, S1_Text.doc, S1_Data.xlsx, S1_PRISMA_Checklist.docx, etc. | Completed. |
| **Production issues 3:** |  |
| 1. Unfortunately, the source for the map used in Figure 1 does not meet our requirements for copyright licensing and CANNOT BE PUBLISHED. Please note that, because all PLOS articles are published under a CC BY license ([creativecommons.org/licenses/by/4.0/](http://creativecommons.org/licenses/by/4.0/)), we cannot publish proprietary maps such as Google Maps, Mapquest or other copyrighted maps. Please remake the figure so that the base map used is from an openly available source. If you are unable to make an open source version of the figure, please remove it. | We removed Figure 1 and modified the corresponding part of the text. |
